# Psychometric Validation and Cultural Adaptation of the Simplified Chinese eHealth Literacy Scale: Cross-Sectional Study

**DOI:** 10.2196/18613

**Published:** 2020-12-07

**Authors:** Richard Huan Xu, Lingming Zhou, Sabrina Yujun Lu, Eliza Laiyi Wong, Jinghui Chang, Dong Wang

**Affiliations:** 1 JC School of Public Health and Primary Care The Chinese University of Hong Kong Hong Kong Hong Kong; 2 School of Health management Southern Medical University Guangzhou China; 3 Department of Sports Science and Physical Education Chinese University of Hong Kong Hong Kong Hong Kong

**Keywords:** electronic health literacy, eHEALS, psychometric property, classical test theory, item response theory, China

## Abstract

**Background:**

The rapid proliferation of web-based information on health and health care has profoundly changed individuals’ health-seeking behaviors, with individuals choosing the internet as their first source of information on their health conditions before seeking professional advice. However, barriers to the evaluation of people’s eHealth literacy present some difficulties for decision makers with respect to encouraging and empowering patients to use web-based resources.

**Objective:**

This study aims to examine the psychometric properties of a simplified Chinese version of the eHealth Literacy Scale (SC-eHEALS).

**Methods:**

Data used for analysis were obtained from a cross-sectional multicenter survey. Confirmatory factor analysis (CFA) was used to examine the structure of the SC-eHEALS. Correlations between the SC-eHEALS and ICEpop capability measure for adults (ICECAP-A) items and overall health status were estimated to assess the convergent validity. Internal consistency reliability was confirmed using Cronbach alpha (α), McDonald omega (ω), and split-half reliability (λ). A general partial credit model was used to perform the item response theory (IRT) analysis. Item difficulty, discrimination, and fit were reported. Item-category characteristic curves (ICCs) and item and test information curves were used to graphically assess the validity and reliability based on the IRT analysis. Differential item functioning (DIF) was used to check for possible item bias on gender and age.

**Results:**

A total of 574 respondents from 5 cities in China completed the SC-eHEALS. CFA confirmed that the one-factor model was acceptable. The internal consistency reliability was good, with α=0.96, ω=0.92, and λ=0.96. The item-total correlation coefficients ranged between 0.86 and 0.91. Items 8 and 4 showed the lowest and highest mean scores, respectively. The correlation coefficients between the SC-eHEALS and ICECAP-A items and overall health status were significant, but the strength was mild. The discrimination of SC-eHEALS items ranged between 2.63 and 5.42. ICCs indicated that the order of categories’ thresholds for all items was as expected. In total, 70% of the information provided by SC-eHEALS was below the average level of the latent trait. DIF was found for item 6 on age.

**Conclusions:**

The SC-eHEALS has been demonstrated to have good psychometric properties and can therefore be used to evaluate people’s eHealth literacy in China.

## Introduction

### Background

Currently, 4 billion people worldwide use the internet for various purposes every day, which increases their ability to search, digest, and use information in every aspect of their lives [1]. The health care field cannot escape from this trend. The rapid proliferation of web-based information about health and health care has significantly changed individuals’ health-seeking behavior, with individuals choosing the internet as their first source of information regarding their health conditions before seeking professional advice [2,3].

Despite increased accessibility to relevant health information on the internet, searching, identifying, and using the *right* information is still challenging. Van der Vaart et al [4] found that easily identifiable web-based information may increase the discrepancies in health knowledge and choice of health care provider. Collecting web-based information is different from acquiring health-related information via traditional methods, such as hospital pamphlets, medical books, or professionals’ advice; acquiring useful and accurate web-based information necessitates specific skills [3,5,6]. These skills include not only having professional knowledge about any given health conditions but also computer literacy, mobile phone literacy, and knowing how to navigate the internet [4,7]. Thus, eHealth literacy, including computer literacy and internet-related knowledge and skills, plays a key role in helping people search for web-based information and analyze, assess, and use that information to improve their health outcomes [8]. eHealth information offers little value if the intended users lack the skills to effectively use this type of information [9]. Measuring people’s eHealth literacy could help policy makers develop guidelines, strategies, and interventions to provide health information through the internet to people regarding different needs, preferences, and expectations in ways that they can understand and use it [7].

In the last two decades, China has made remarkable progress in the development of internet networks and services. Currently, more than 800 million Chinese people actively use the internet every day [10]. To maximize the effectiveness of the internet in improving people’s health, in 2018, the State Council of China announced an ambitious plan to develop an integral system to provide a broad spectrum of health care services through the internet across China in the next 10 years; this initiative intends to solve the long-term problems of *kanbing nan and kanbing gui* (too inaccessible and expensive to visit physicians) [11]. An instrument that comprehensively evaluates people’s skills and literacy to understand and use web-based information and services is essential. In particular, given that China has a dual social urban-rural system, the gap between the cities and the countryside, including economic, educational, health care, and so on, was and continues to be tremendous. The provision of consumer-tailored eHealth information that meets their levels of eHealth literacy is the key to engaging them in making sound health decisions [9].

At present, few measures exist to specifically measure eHealth literacy, which has a different conceptual structure compared with traditional health literacy. In 2006, the eHealth literacy scale (eHEALS) was developed to focus on assessing the skills for finding, evaluating, and applying web-based health information to improve one’s health outcomes [12]. It has been translated and validated in several countries and has been demonstrated to be a valuable instrument for assessing the effects of eHealth literacy on helping decision makers develop appropriate strategies to provide web-based information to improve people’s health.

In China, several studies have reported the performance of eHEALS in different settings, and all of them showed some limitations. The generalizability of the findings from the study by Chang and Schultz [13] was questionable because they used a total of 352 patients from one selected hospital from a city in China, and their targeted populations were only those who reported having chronic conditions. For the study by Ma and Wu [14], the major limitation was that all the respondents included in that study were rural residents and were recruited from one district of a small city in Western China (the poorest part of China). By 2018, approximately 60% of the population in China lived in urban areas; therefore, assessing the psychometric properties of the eHEALS on only rural residents from one district would lead to ubiquitous selection and information bias [14]. Furthermore, the study by Zibrik et al [15] also reported the psychometric properties of the eHEALS; however, their study was not conducted in mainland China, and the targeted population was British Columbia’s immigrant Chinese and Punjabi senior population. The study by Guo et al [16] seemed to be the first psychometric study of the Chinese eHEALS; however, 2 issues need to be addressed. First, their work was presented in Chinese rather than English, where the results are difficult for non-Chinese researchers to understand—this limits international or regional comparisons within Asian countries or between Chinese populations from different countries. Second, the targeted population of that study was 110 high school students recruited from Beijing (the most developed region in China). The selection and information bias could not be neglected. Moreover, except for the study by Ma and Wu [14], no previous studies used both classical test theory (CTT) and item response theory (IRT) to assess the validity of the Chinese eHEALS, and no study has adopted the differential item functioning (DIF) analysis to investigate the item variance of the scale. Considering the limitations summarized above, it is important to assess the performance of the Chinese version of eHEALS using more comprehensive methods and a better representative sample, which covers respondents from developed and underdeveloped areas, urban and rural residents, and across a wide age range.

### Aims of the Study

This study aims to examine the psychometric properties of a simplified Chinese version of the eHEALS (SC-eHEALS) in the Chinese population based on a multicenter cross-sectional study.

## Methods

### Translation

The translation of the SC-eHEALS followed the international protocol [17]. Two translators, who were native Chinese speakers but were fluent in English, were invited to translate the eHEALS from English into simplified Chinese independently. The 2 transcripts were collected by the local research team for discussion and synthesis. Subsequently, a revised version was produced and sent to 2 professional translators for independent backward translation. The local research team examined the back translation against the original English version to identify any discrepancies, addressed the disputed items, and refined the translation, focusing on cultural adaptation until consensus was achieved by all the research team members. Then, a cognitive debriefing was carried out that involved 15 patients recruited from a hospital in Guangzhou. All the respondents were asked to comment on the response options and any word or phrasing that they found difficult to understand. Respondents were asked to describe in their own language what the word or phrasing meant to them. The results of the pilot study were discussed by the local research team, and then the final version of the SC-eHEALS was verified.

### Study Design and Data Collection

Data used in this study were obtained from a multicenter cross-sectional survey that investigated patients’ attitudes toward patient-centered care (PCC) in China from November 2019 to January 2020. Patients were recruited from the inpatient departments of 8 hospitals from 5 cities (Guangzhou, Shenzhen, Zhanjiang, Meizhou, and Shaoguan) of the Guangdong province (Southern China). All patients from the target hospitals during the appointed survey period were invited to participate in the survey. The inclusion criteria were as follows: (1) aged ≥18 years, (2) can read and speak Chinese, (3) no cognitive problems, and (4) able to provide informed consent. A team of investigators with rich experience in conducting face-to-face interviews was recruited to carry out the fieldwork. With the assistance of ward nurses, all respondents were asked to complete a structured questionnaire that included questions about demographic characteristics, socioeconomic status (SES), health conditions, well-being, health services use, lifestyle, and attitudes toward PCC. To conduct confirmatory factor analysis (CFA), the minimum sample size required was 300 [18,19]. For the IRT analysis, the preferred sample size required is 500 for analyzing a scale comprising polytomous items [20]. In total, the data of 574 patients who completed the SC-eHEALS questionnaire were elicited for our psychometric analysis. Informed consent was obtained from all respondents. The study protocol was approved by the institutional review board of the second affiliated hospital of Guangzhou Medical University (ethical approval ID: 2019-ks-28).

### Instrument

#### eHEALS

eHEALS is used to measure consumers’ combined knowledge, comfort, and perceived skills at finding, evaluating, and applying eHealth information to manage health problems [12]. It was developed based on a framework that consists of 6 dimensions to understand and use eHealth information [9]. The eHEALS has 8 items that are rated on a 5-point Likert scale ranging from *strongly disagree* to *strongly agree*. A one-factor structure was confirmed in the original study, and the reliability was acceptable with a Cronbach alpha of .88 [12]. The sum score of eHEALS ranges from 8 to 40, where a higher score indicates greater perceived eHealth literacy. The translation of eHEALS to SC-eHEALS has been discussed earlier.

#### The ICEpop CAPability Measure for Adults

The ICEpop capability measure for adults (ICECAP-A) is a generic and preference-based instrument that evaluates an individual’s well-being [21]. It has 5 items (stability, attachment, autonomy, achievement, and enjoyment), and each item has 4 response options that range from fully capable to not capable.

#### Overall Health Status

The respondents’ overall health status was evaluated using a visual analogue scale (VAS). They were presented with a scale ranging from 0 to 100, where 0 represents the worst health status and 100 represents the best health status they can imagine on the surveying day.

### Statistical Analysis

#### Construct Validity

CFA was used to examine the structure of SC-eHEALS. The model fit was determined by 4 indicators, that is, root mean square error of approximation (RMSEA≤0.08, fair), the comparative fit index (CFI>0.95, good), the Tucker-Lewis index (TLI>0.95, good), and standardized root mean square residual (SRMR<0.08, acceptable) [22]. Moreover, the Akaike information criterion (AIC) and Bayesian information criterion (BIC) were supplemented to compare the performance of the models, with smaller values indicating a better fit. Exploratory factor analysis (EFA) was used when the one-factor model reported in the original study was not supported by CFA.

#### Convergent Validity

Convergent validity was confirmed by evaluating the correlations between the SC-eHEALS and ICECAP-A items and the VAS item. Pearson correlation coefficient (r) was used to assess the strength of the correlation with r>0.3 identified as *moderate* [23].

#### Known-Group Validity

One-way analysis of variance (ANOVA) was used to assess the known-group validity of the SC-eHEALS. As individuals’ levels of eHealth literacy were impossible to observe directly, on the basis of literature review, we hypothesized that the patients who were young, highly educated, or fully employed (representing good SES) would show a high degree of eHealth literacy.

#### Item Statistics and Internal Consistency Reliability

The mean and SD of the SC-eHEALS scores at both the item- and scale-level were reported as well as the ceiling and floor effects. The internal consistency reliability was estimated using Cronbach alpha (α>.7, acceptable), McDonald omega (ω>0.7, acceptable), and split-half reliability (λ>0.7, acceptable). Item-total correlations (>0.7 acceptable) and alpha were presented if items were deleted [23].

#### IRT Analysis

Considering that there is no gold standard for model selection to perform IRT analysis, we adopted 3 models in this study, compared their performance, and reported the results of the best-fit model. The first model was the partial credit model (PCM), which is an extended version of the Rasch model. The second model was the general partial credit model (GPCM), which is a modified version of PCM and used to estimate 2 parameters in the analysis; and the last was the rating scale model, which is another Rasch family model that requires all items to have the same number of options. The details of the IRT models can be found in the study by DeMars [24]. The likelihood ratio test was used to compare the performance of the models. AIC and BIC were also used to assess the model fit. The results of model comparisons showed the GPCM outperformed the other 2 models [25]. For GPCM analysis, the (1) discrimination, (2) difficulty, and (3) item fit (the value of S-χ2) were calculated for each item, along with the item information curves (IICs) and test information curve (TIC) [26]. The item-category characteristic curves (ICCs) graphically presented the probability of the response to a given item in each category as the function of the latent trait (eHealth literacy).

#### Differential Item Functioning

DIF was used to check for the possible item bias caused by responses from different subgroups (gender, age, family registry, chronic conditions, and educational level) in the sample [27]. Pseudo McFadden *R*^2^ was used to estimate the magnitude of the DIF. *R*^2^<0.13 was deemed as *negligible*, 0.13 to 0.26 as *moderate*, and >0.26 as *large*. [28]

R (R foundation) was used for data analysis, and the *P* value was set at <.05. For IRT analysis, the Bonferroni correction was applied, and the *P* value was set at <.006 (.05/8).

## Results

### Respondents’ Characteristics

In this study, more than half of the respondents were men (292/574, 50.9%), and the mean age was 45.58 years (SD 16.18). Almost half of them completed tertiary educational attainment (263/574, 45.8%), and 50.8% (231/574) were urban residents. Nearly half of the respondents reported living with chronic conditions (281/574, 49.1%) and only one fifth of the respondents reported that their disease was a severe threat to their lives (112/574, 19.8%; Table 1).

**Table 1:** Respondents’ characteristics (n=574).

Characteristic		Value
**Sex, n (%)**		
	Male	292 (50.9)
	Female	281 (48.9)
Age (years), mean (SD)		45.58 (16.18)
**Education, n (%)**		
	No or primary	93 (16.2)
	Secondary or postsecondary	218 (37.9)
	Tertiary or above	263 (45.8)
**Family registry, n (%)**		
	Urban	291 (50.8)
	Rural	282 (49.2)
**Caregiver, n (%)**		
	No	418 (72.8)
	Yes	156 (27.2)
**Living status, n (%)**		
	Live alone	57 (9.9)
	Live with family or others	517 (90.1)
**Working status, n (%)**		
	Employed	394 (68.6)
	Unemployed	180 (31.4)
**Income level^a^(CNY ¥ [US $]), n (%)**		
	≤900^b^ (64.3)	112 (19.6)
	901-1800 (64.4-128.6)	48 (8.4)
	1801-2700 (128.6-192.9)	68 (11.9)
	2701-3800 (192.9-271.4)	78 (13.7)
	3801-6400 (271.4-457.1)	125 (21.9)
	≥6401 (457.1)	140 (24.5)
**BMI, n (%)**		
	<23	294 (51.7)
	≥23	275 (48.3)
**Chronic condition, n (%)**		
	Yes	281 (49.1)
	No	291 (50.9)
**Self-reported health condition, n (%)**		
	Severe threat to life	112 (19.8)
	Moderate threat to life	112 (19.8)
	Mild threat to life	137 (24.2)
	No threat to life	206 (35.8)

^a^Disposable personal income per month.

^b^100 CNY ¥ equals approximately 14 US $ (February 2020 exchange rate).

### Construct Validity

The results of the CFA showed that the model fit of the one-factor model was not satisfactory with a low TLI and high RMSEA values (Table 2). We further examined items with high residual correlations and modification indices. On examining the wordings and polychoric correlation of items, we found that the nonrandom measurement error was caused by item redundancy. The fit of the revised model was improved after we modified the model and specified the error covariance between items 1 and 2, 3 and 4, and 7 and 8. A two-factor structure was suggested by the EFA (Multimedia Appendix 1). The CFA indicated that the performance of the revised two-factor model was not better than the revised one-factor model. After checking the item correlations and residuals, a revised two-factor model was confirmed, with RMSEA=0.08, SRMR=0.02, CFI=0.99, and TLI=0.96. The revised two-factor model outperformed the other models. The standardized factor loadings for the observed variables of all CFA models are presented in Multimedia Appendix 2.

**Table 2:** Confirmatory factor analysis of the simplified Chinese eHealth literacy scale.

Model	Model fit statistics							
	χ^2^ (*df*)	*P* value	RMSEA^a^ (95% CI)	CFI^b^	TLI^c^	SRMR^d^	AIC^e^	BIC^f^
One-factor	443.56 (20)	<.001	0.19 (0.18-0.21)	0.92	0.88	0.04	7905.29	7974.93
Revised one-factor	134.76 (17)	<.001	0.11 (0.09-0.12)	0.97	0.96	0.03	7602.49	7685.19
Two-factor	252.73 (19)	<.001	0.15 (0.13-0.16)	0.95	0.93	0.03	7716.46	7790.45
Revised two-factor	83.2 (17)	<.001	0.08 (0.06-0.1)	0.99	0.98	0.02	7550.93	7633.63

^a^RMSEA: root mean square error of approximation.

^b^CFI: comparative fit index.

^c^TLI: Tucker-Lewis index.

^d^SRMR: standardized root mean square residual.

^e^AIC: Akaike information criterion.

^f^BIC: Bayesian information criterion.

### Convergent and Known-Group Validity

Table 3 shows the correlations between the SC-eHEALS and ICECAP-A items and overall health status. All the correlations of SC-eHEALS with the other measures were statistically significant, but the value of coefficients indicated a mild correlation. The results of ANOVA indicate that respondents who were young, highly educated, and fully employed tended to report a high level of eHealth literacy, which indicated that the known-group validity of the SC-eHEALS was satisfied (Table 4).

**Table 3:** Convergent validity of the simplified Chinese eHealth literacy scale.

Simplified Chinese eHealth literacy scale items^a^	ICECAP-A^b^										VAS^c^		
	Stability	*P* value	Attachment	*P* value	Enjoyment	*P* value	Achievement	*P* value	Autonomy	*P* value		Value	*P* value
eHEALS^d^ 1	−0.15	<.001	−0.16	<.001	−0.12	.003	−0.11	.007	−0.17	<.001		0.12	.006
eHEALS 2	−0.13	.002	−0.14	.001	−0.12	.003	−0.13	.002	−0.12	.004		0.11	.007
eHEALS 3	−0.16	<.001	−0.19	<.001	−0.18	<.001	−0.18	<.001	−0.23	<.001		0.10	.02
eHEALS 4	−0.15	<.001	−0.14	<.001	−0.15	<.001	−0.11	.01	−0.20	<.001		0.10	.02
eHEALS 5	−0.17	<.001	−0.15	<.001	−0.15	<.001	−0.14	.001	−0.17	<.001		0.09	.05
eHEALS 6	−0.16	<.001	−0.20	<.001	−0.14	<.001	−0.14	.001	−0.19	<.001		0.11	.007
eHEALS 7	−0.18	<.001	−0.13	<.001	−0.17	<.001	−0.18	<.001	−0.15	<.001		0.17	<.001
eHEALS 8	−0.20	<.001	−0.17	<.001	−0.18	<.001	−0.16	<.001	−0.17	<.001		0.14	.001
Sum score	−0.18	<.001	−0.19	<.001	−0.16	<.001	−0.16	<.001	−0.20	<.001		0.14	.001

^a^For the eHealth literacy scale, a higher score indicates good eHealth.

^b^For ICECAP-A, a higher score indicates worse status of well-being.

^c^VAS: visual analogue scale of overall physical health.

^d^eHEALS: eHealth literacy scale.

**Table 4:** Known-group validity of the simplified Chinese eHealth literacy scale.

Characteristics		Mean (SD)		*P* value
**Age (years)**			.003	
	≤30	30.85 (6.28)		
	31-60	29.34 (6.42)		
	≥61	27.28 (7.91)		
**Education**			.02	
	No or primary	28.29 (7.99)		
	Secondary or postsecondary	28.46 (7.05)		
	Tertiary or above	30.27 (5.93)		
**Working status**			.003	
	Employed	29.95 (6.46)		
	Unemployed	27.75 (7.22)		

### Item Statistics and Internal Consistency Reliability

Table 5 shows that item 8 was rated as the most difficult item with a mean score of 3.49 (SD 1.01), whereas item 4 was rated as the easiest item with a mean score of 3.75 (SD 0.93). Items showed no floor effects, but some ceiling effects, which ranged from 16.9% to 19.69%. The reliability of the scale was good, as the Cronbach alpha was .96, McDonald omega was 0.92 and split-half reliability was 0.96. In addition, the value of alpha if an item was deleted was lower than that observed if the item was retained, and the item-total correlation coefficients ranged between 0.86 and 0.91. The SC-eHEALS scores stratified according to respondents’ characteristics are presented in Multimedia Appendix 3.

**Table 5:** The response pattern, item statistics, and internal consistency reliability of the simplified Chinese eHealth literacy scale.

SC-eHEALS^a^	Response, n						Item statistics				Internal consistency reliability	
	Strongly disagree	Disagree	Neutral	Agree	Strongly agree	Mean (SD)		Floor, n (%)	Ceiling, n (%)	Alpha if item deleted^b^		Item-total correlation^b^
eHEALS^c^ 1	18	36	150	267	103	3.70 (0.94)		18 (3.14)	103 (17.94)	.88		0.91
eHEALS 2	16	29	156	264	109	3.73 (0.92)		16 (2.79)	109 (18.99)	.88		0.91
eHEALS 3	14	39	141	278	102	3.72 (0.92)		14 (2.44)	102 (17.77)	.82		0.86
eHEALS 4	15	37	135	274	113	3.75 (0.93)		15(2.61)	113(19.69)	.86		0.89
eHEALS 5	17	49	160	242	106	3.65 (0.97)		17 (2.96)	106 (18.47)	.87		0.90
eHEALS 6	17	45	169	242	101	3.64 (0.96)		17 (2.96)	101 (17.60)	.86		0.89
eHEALS 7	20	47	195	208	104	3.57 (0.99)		20 (3.48)	104 (18.12)	.84		0.88
eHEALS 8	22	58	205	192	97	3.49 (1.01)		22 (3.83)	97 (16.90)	.81		0.86
Overall	N/A^d^	N/A	N/A	N/A	N/A	29.26 (6.78)^e^		8 (1.57)	40 (10.63)	N/A		N/A

^a^SC-eHEALS: simplified Chinese eHealth literacy scale.

^b^Cronbach alpha=.96; McDonald omega=.92; split-half reliability=.96.

^c^eHEALS: eHealth literacy scale.

^d^N/A: not applicable.

^e^The overall score of the eHEALS ranges between 0 and 40.

### IRT Analysis

The results of the GPCM analysis are presented in Table 6. The discrimination indices of items ranged between 2.63 and 5.42, which indicated that the SC-eHEALS can distinguish between individuals with either low or high eHealth literacy, corresponding with the latent trait sensitivity. The item difficulty of threshold 1 and threshold 4 ranged from −2.03 to −1.79 and 0.84 to 1.04, respectively. The *P* value of S-χ^2^ (Specific item-fit index) reflected that items 3 (*P*<.001), 4 (*P*=.003), and 8 (*P*<.001) might show misfit to some extent. Most of the information (70%) provided by the SC-eHEALS was between −6 and 0 on the latent trait scale (the comparisons between different IRT models are presented in Multimedia Appendix 4, and the misfit items of the SC-eHEALS are presented in Multimedia Appendix 5).

**Table 6:** The result of item response theory analysis of the simplified Chinese eHealth literacy scale.

SC-eHEALS^a^	Discrimination of item	Difficulty of item				Specific item-fit index, S-χ^2^ (df)		*P* value^b^		Information (0-100^c^)		
		T1^d^	T2^e^	T3^f^	T4^g^						−6 to 0	0 to 6
eHEALS 1	5.27	−1.79	−1.27	−0.41	0.90		19.041 (17)		.33		72.65	27.35
eHEALS 2	5.42	−1.85	−1.37	−0.42	0.84		24.171 (16)		.09		72.95	27.05
eHEALS 3	3.09	−2.03	−1.37	−0.51	1.01		61.57 (21)		<.001		71.62	28.38
eHEALS 4	4.17	−1.94	−1.31	−0.50	0.85		38.969 (18)		.003		72.95	27.05
eHEALS 5	4.52	−1.87	−1.17	−0.33	0.89		32.962 (19)		.05		70.73	29.27
eHEALS 6	3.83	−1.89	−1.24	−0.31	0.96		27.916 (20)		.11		69.67	30.33
eHEALS 7	3.24	−1.80	−1.26	−0.17	0.95		41.64 (24)		.01		66.58	33.42
eHEALS 8	2.63	−1.80	−1.20	−0.06	1.04		73.951 (25)		<.001		64.01	35.99
Overall	N/A^h^	N/A	N/A	N/A	N/A		N/A		N/A		70.70	29.30

^a^SC-eHEALS: simplified Chinese eHealth literacy scale.

^b^Items with *P*<.05/8=.0062 were flagged as potentially misfit.

^c^0 means no information, 100 means full information.

^d^T1: threshold categories 1 and 2.

^e^T2: threshold categories 2 and 3.

^f^T3: threshold categories 3 and 4.

^g^T4: threshold categories 4 and 5.

^h^N/A: not applicable.

**Figure 1:**
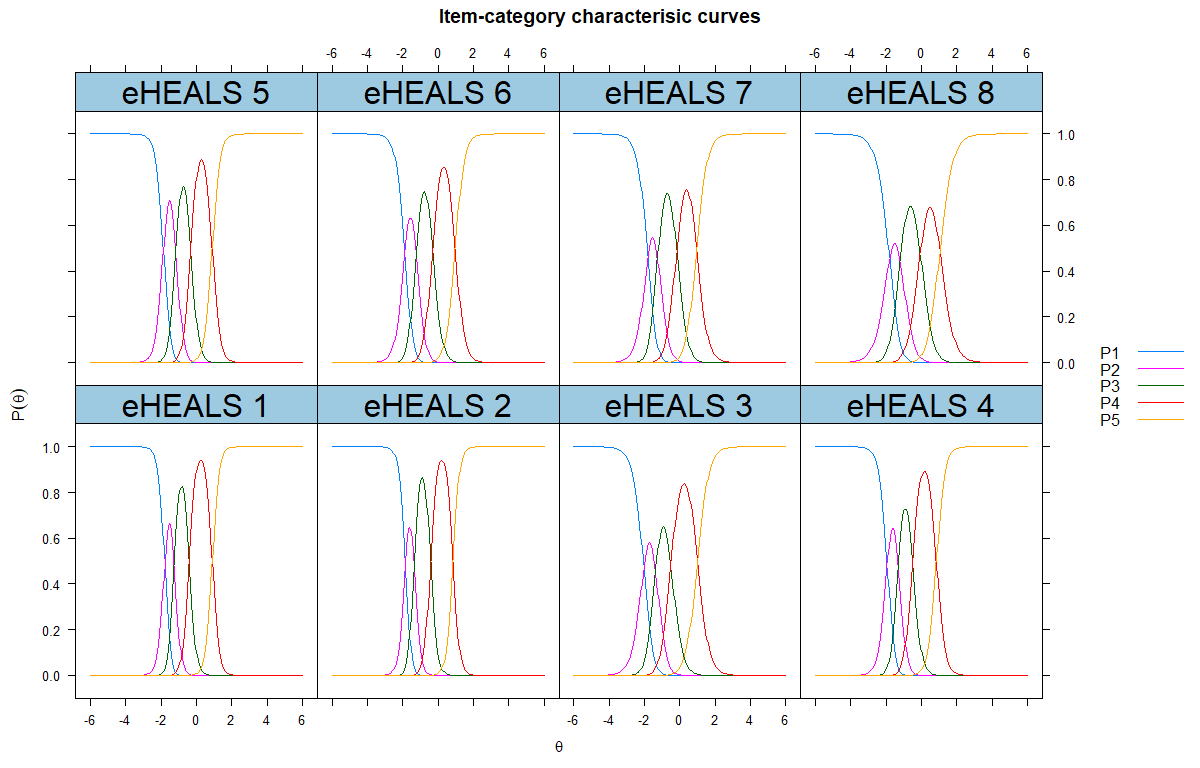
Item-category characteristic curves for the simplified Chinese eHealth literacy scale. eHEALS: eHealth literacy scale; P: probability of option to be selected.

**Figure 2:**
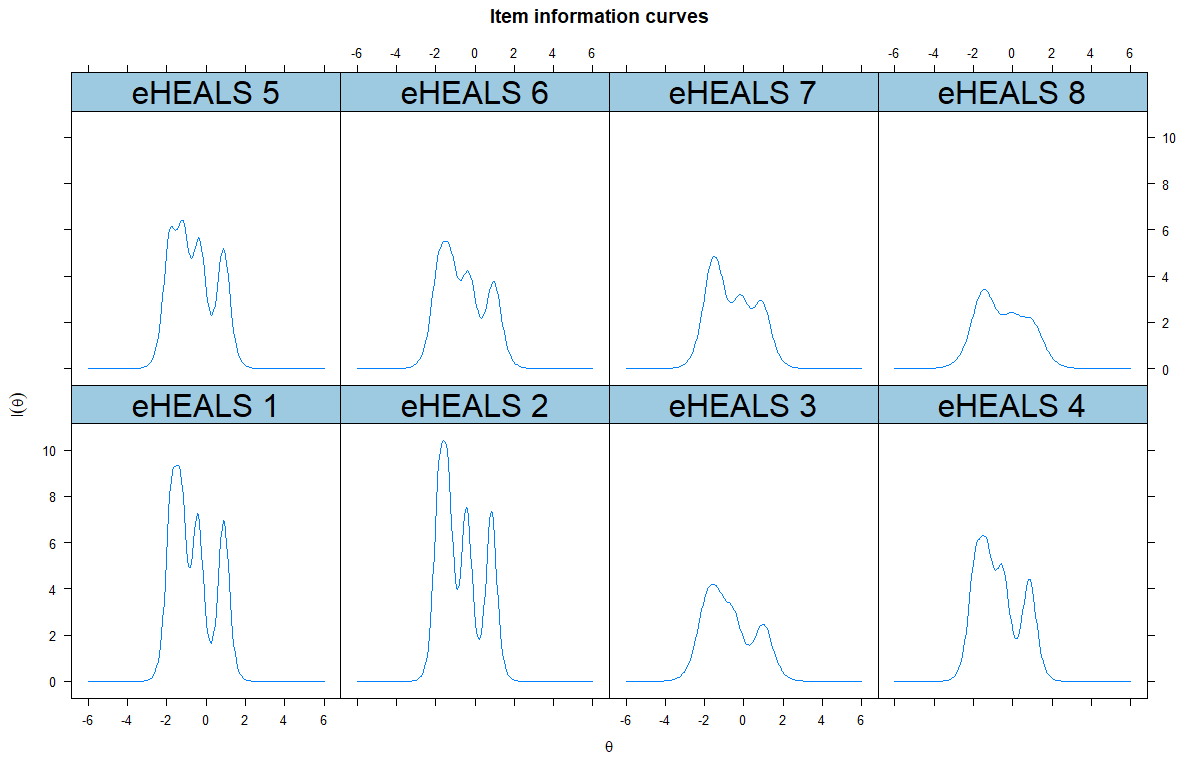
The item information curves for items of the simplified Chinese eHealth literacy scale. eHEALS: eHealth literacy scale.

The ICCs and IICs for SC-eHEALS are graphically presented in Figures 1 and 2, respectively. The curves of the ICCs showed that the order of categories’ thresholds for all the items was as expected, which meant that all categories were adequate in terms of placing a respondent on the scale. The distributions of the IICs were multimodal. The shapes of items 1 and 2 were the steepest and provided more information than the other items. The shapes of items 3 and 8 were the flattest, which means little information was provided by these items. TIC of the SC-eHEALS is presented in Multimedia Appendix 6.

**Figure 3:**
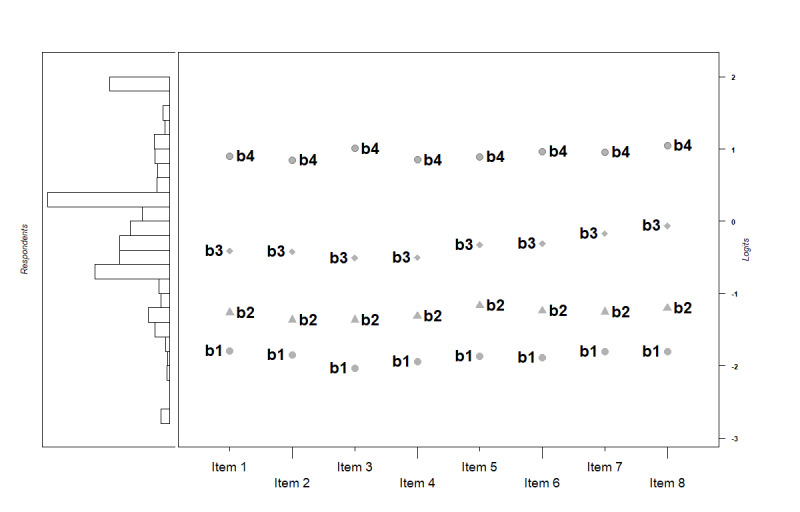
Item-person map of the simplified Chinese eHealth literacy scale. b: coefficient of threshold.

Item-person map displays the location parameters of items as well as the distribution of person parameters along the same latent dimension. We found that the gap between threshold 3 and threshold 4 was larger than the other gaps; however, the discrepancy was diminished from item 1 to 8. In contrast, the gap between threshold 1 and threshold 2 was smaller than the others; however, the discrepancy increased from items 1 to 8. The distribution of person parameters showed some respondents located at the high end of the latent trait scale, which means they are very likely equipped with strong eHealth literacy (Figure 3).

### DIF Analysis

Item 6 of the SC-eHEALS showed a uniform DIF when considering the respondents from different age groups. However, the magnitude was smaller than 0.13, confirming that the effect size of the DIF was negligible [29] (Multimedia Appendix 7).

## Discussion

### Principal Findings

In China, an increasing number of people have turned to the internet to seek health care information because of the rapid proliferation of web-based medical information. However, whether users can leverage such information to improve their health can hardly be measured. The findings of this study support that the SC-eHEALS (Multimedia Appendix 8) is a valid and reliable instrument for measuring self-reported eHealth literacy in China. For the general public, the SC-eHEALS provides a measure to help them evaluate web-based health information and critically appraise the quality of eHealth resources, which could protect them from the harm of low-quality information and empower them to manage their health. Policy makers provide important information for understanding the population’s perceived degree of eHealth literacy and developing cost-effective strategies for upgrading the medical care sector by leveraging the internet [30].

### Comparisons With Previous Studies

The original one-factor structure of the eHEALS, as confirmed by previous studies in mainland China [13,14], was acceptable, but was not fully supported by our study. This was not an unforeseen result; some other studies have reported a two-factor [4,31] or a three-factor structure [3,32]. Diviani et al [33] pinpointed that when using CFA, neither the single-factor model, originally proposed by the developers, nor the two-factor model, suggested by other research, showed an adequate fit to the data. Cummings [34] indicated that translation might change the original meaning of the items, and these changes could affect perceived meanings for targeted respondents. Furthermore, the original eHEALS was developed at a time before the rise of social media, which totally changed people’s interaction with health information, which might affect the structure of the eHEALS [32]. Furthermore, the eHEALS was developed based on a model that originally consisted of 6 domains of literacy, and Noman et al [12] suggested that each skill would require independent measurement. However, in this study, the two-factor structure was not without problems. First, the values of the CFI, TLI, and SRMR indicated that the performance of the revised one-factor model was not worse than that of the two-factor model. Only the RMSEA value supported the revised two-factor model outperforming the other models. A similar finding was reported in the study by Paige et al [3], in which they finally confirmed the one-factor structure. Second, the factor loadings of each item in the revised two-factor model were similar to the one-factor model; item 7 even showed a lower value (0.594), which indicated that the difference in stability between the 2 structures was negligible. Given that people’s different abilities to manipulate web-based information is attributed to their demographics, SES, and health conditions, we decided to maintain the one-factor structure. Studies exploring the structure of the SC-eHEALS in other subpopulations should be carried out in the future. A clear structure of the SC-eHEALS would be useful in facilitating the computer adaptive test (CAT) in future practice. Administering the SC-eHEALS based on CAT can strengthen its precision and sensitivity [3], which ensures that the chosen items can meet the respondents’ eHealth literacy levels.

The proportion of respondents choosing the options of *neutral* and above was high in this study, which might indicate that most of them are equipped with middle-to-high eHealth literacy and skills and are confident in searching, understanding, analyzing, and using eHealth information. The ceiling effect of all the items of the SC-eHEALS was detected, despite the strength of the effect not being very strong. The distribution of the responses in eHEALS was not reported by other studies in China. However, Paige et al [3] reported that the mean score of the eHEALS ranged between 3.57 and 3.96 in the US population. It remained that the discriminant ability of the SC-eHEALS might not be strong enough to differentiate people with different levels of eHealth literacy. In addition, the SC-eHEALS showed an excellent reliability with a Cronbach alpha (.96) higher than that reported by other studies conducted in China [13,14] and for some other language versions [3,4,12,35,36]. However, Chang and Schultz [13] found that removing items 7 or 8 could improve the reliability of the eHEALS in China, which was not reported by any other study. Moreover, we found that the mean score of the eHEALS varied widely across different populations. For example, Diviani et al [33] reported the same findings as ours, as respondents rated item 4 as the easiest and item 8 as the most difficult. Chung and Nahm [36] found that item 4 was perceived as the easiest item, whereas item 7 was perceived as the most difficult item by a US sample. Van der Vaart et al [4] identified that items 3 and 8 received the highest and lowest mean scores of all items, respectively. Given that different studies have recruited samples with different subpopulations and that the proliferation of the internet in different countries is varied, further exploration should be carried out to draw more international comparisons.

The GPCM was demonstrated to be the best-fit IRT model in this study. No previous studies used the GPCM to test the psychometric properties of eHEALS. Diviani et al [33] used the nonparameter IRT model, Paige et al [3] used the PCM model, and Zrubka et al [35] and Ma and Wu [14] used the graded response model. Our study enriches the knowledge of the application of IRT models and supports existing research on the psychometric analysis of eHEALS using IRT methods. GPCM analysis showed that the overall performance of SC-eHEALS was satisfactory. ICCs indicated that the response categories of each item were ordered, and all categories were most probably at the same point on the continuum. For the location parameter, all items were placed between −3 and 3 across the scale of the latent trait. The discrimination of the items was positive and could discriminate between individuals with different levels of eHealth literacy. The item-person map demonstrated that the items of the SC-eHEALS were located within a reasonable range and were well centered with respect to the person measure distribution. Diviani et al [33] reported similar findings in the Italian eHEALS; however, Paige et al [3] showed that the response categories of the eHEALS covered a wider range of latent traits. The information curves showed a multimodal distribution of rural Chinese citizens [14] and the Hungarian population as shown in the study by Zrubka et al [35]. The TIC of the SC-eHEALS provided the precise estimation near the center of the ability scales, which ranged between −3 and 2, but as the ability level approached the extremes of the scale, the accuracy of the test decreased significantly. The plot of misfit items showed that the majority of imprecise estimations occurred at the options of *neutral* and agree, indicating that these 2 options might not be accurate enough to measure the latent trait among people with moderate-to-high eHealth literacy. To yield a more precise measure of eHealth literacy, future studies should explore what response options are more appropriate to be included in the SC-eHEALS [4].

Moreover, item 6 (skills to evaluate health resources) was labeled as having DIF among respondents of different age groups (older vs younger), which reflected that they have an unequal probability of giving a response to this item. No previous study reported a similar finding at the item level using DIF analysis. Ma and Wu [14] indicated that item 6 has the lowest discriminant power in Chinese eHEALS. However, caution should be exercised when drawing this conclusion, as we cannot determine whether the DIF occurred due to a form of measurement error [37]. In other words, we can neither unequivocally infer that the DIF of item 6 reflected real group differences of the underlying trait among respondents nor attribute this finding to bias during the process of measurement.

### Strengths and Limitations

This study has several strengths. First, the respondents who participated in this study came from both developed and underdeveloped areas, were urban and rural residents, and spanned a wide age range, showing better representativeness than the previous studies in China. Second, this study assessed the psychometric properties of SC-eHEALS using both CTT and IRT methods. Third, for IRT analysis, the best-fit model was selected based on comparison of the performance of 3 IRT models. Fourth, DIF analysis was first used to evaluate the item bias and variance of the eHEALS in the Chinese population. The comparisons between the characteristics of the Chinese eHEALS validation studies are presented in Multimedia Appendix 9.

Several limitations of this study should be addressed. First, all respondents were from inpatient departments. This means that most of them might have poor health status, which posed some degree of selection bias. Patients with mild or no health problems should be included in follow-up studies to further test the psychometric properties of SC-eHEALS. Second, the sample size of this study was less than 1000, which might generate some uncertainties in estimating the IRT model, especially using the GPCM (a two-parameter model). A larger sample should be collected in future studies to validate our findings. Third, we did not differentiate between patients with different diseases when evaluating the psychometric properties of the SC-eHEALS, which might have created some problems in explaining the validity of the instrument. The performance of SC-eHEALS should be further assessed in distinct patient groups. Fourth, we did not collect information on respondents’ behaviors related to their internet use, such as the frequency and types of websites; thus, criterion validity cannot be assessed. Finally, the information of patients who refused to participate in the survey was not recorded, which might have led to a measure of information bias.

### Conclusions

This study evaluated the psychometric properties of SC-eHEALS using a large sample of patients from a multicenter study in China. It provides empirical evidence that SC-eHEALS is a valid, reliable, and parsimonious instrument for evaluating the degree of eHealth literacy in Chinese people with different demographics, SES (eg, rural and urban residents), and health status. CFA did not fully support the original one-factor structure, and further exploration is needed. IRT analysis suggested that SC-eHEALS might not be effective for use in people with very high or low eHealth literacy. Further studies are needed to detect the heterogeneity of the SC-eHEALS in different subpopulations and further assess its criterion validity.

## Appendix

Multimedia Appendix 1
The results of the exploratory factor analysis.

Multimedia Appendix 2 The standardized factor loadings for the observed variables of all confirmatory factor analysis models.

Multimedia Appendix 3 The simplified Chinese eHealth literacy scale scores stratified according to respondents’ characteristics.

Multimedia Appendix 4 The comparisons between different item response theory models.

Multimedia Appendix 5 The misfit items of the simplified Chinese eHealth literacy scale.

Multimedia Appendix 6 Test information curve of the simplified Chinese eHealth literacy scale.

Multimedia Appendix 7 Results of differential item functioning analysis.

Multimedia Appendix 8 The Chinese version of the eHealth literacy scale.

Multimedia Appendix 9
Summary of the Chinese eHEALS studies.

## Abbreviations

AIC: Akaike information criterion
ANOVA: one-way analysis of variance
BIC: Bayesian information criterion
CAT: computer adaptive test
CFA: confirmatory factor analysis
CFI: comparative fit index
CTT: classical test theory
DIF: differential item functioning
EFA: exploratory factor analysis
eHEALS: eHealth literacy scale
GPCM: general partial credit model
ICC: item-category characteristic curve
ICECAP-A: ICEpop capability measure for adults
IIC: item information curve
IRT: item response theory
PCC: patient-centered care
PCM: partial credit model
RMSEA: root mean square error of approximation
SC-eHEALS: simplified Chinese eHealth literacy scale
SES: socioeconomic status
SRMR: standardized root mean square residual
TIC: test information curve
TLI: Tucker-Lewis index
VAS: visual analogue scale
