# Supplementary material for: Psychometric Validation and Cultural Adaptation of the Simplified Chinese eHealth Literacy Scale: Cross-Sectional Study
Source: J Med Internet Res. 2020 Dec 7;22(12):e18613. doi: 10.2196/18613 (PMC7752540; doi:10.2196/18613)
Supplement: Multimedia Appendix 1 [file jmir_v22i12e18613_app1.docx]

**The result of EFA**

|  | Factor loadings |
| --- | --- |
| One-factor model |  |
| eheals1 | 0.9 |
| eheals2 | 0.9 |
| eheals3 | 0.84 |
| eheals4 | 0.88 |
| eheals5 | 0.89 |
| eheals6 | 0.88 |
| eheals7 | 0.86 |
| eheals8 | 0.82 |
|  |  |
| Two-factor model |  |
| F1 |  |
| eheals1 | 0.74 |
| eheals2 | 0.69 |
| eheals3 | 0.94 |
| eheals4 | 0.97 |
| eheals5 | 0.49 |
| F2 |  |
| eheals6 | 0.65 |
| eheals7 | 0.82 |
| eheals8 | 0.96 |
