# Supplementary material for: Psychometric Validation and Cultural Adaptation of the Simplified Chinese eHealth Literacy Scale: Cross-Sectional Study
Source: J Med Internet Res. 2020 Dec 7;22(12):e18613. doi: 10.2196/18613 (PMC7752540; doi:10.2196/18613)
Supplement: Multimedia Appendix 2 [file jmir_v22i12e18613_app2.docx]

**Standardized factor loadings of CFA models**

|  | Factor loading | Standard error | p-value |
| --- | --- | --- | --- |
| **One-factor model** |  |  |  |
| eheals1 | 0.905 | 0.009 | <0.001 |
| eheals2 | 0.905 | 0.009 | <0.001 |
| eheals3 | 0.843 | 0.013 | <0.001 |
| eheals4 | 0.883 | 0.01 | <0.001 |
| eheals5 | 0.892 | 0.01 | <0.001 |
| eheals6 | 0.872 | 0.011 | <0.001 |
| eheals7 | 0.848 | 0.013 | <0.001 |
| eheals8 | 0.815 | 0.015 | <0.001 |
|  |  |  |  |
| **Revised one-factor model** |  |  |  |
| eheals1 | 0.897 | 0.01 | <0.001 |
| eheals2 | 0.898 | 0.01 | <0.001 |
| eheals3 | 0.82 | 0.015 | <0.001 |
| eheals4 | 0.866 | 0.012 | <0.001 |
| eheals5 | 0.902 | 0.009 | <0.001 |
| eheals6 | 0.882 | 0.011 | <0.001 |
| eheals7 | 0.841 | 0.013 | <0.001 |
| eheals8 | 0.809 | 0.016 | <0.001 |
| eheals1~eheals2 | 0.227 | 0.051 | <0.001 |
| eheals3~eheals4 | 0.516 | 0.034 | <0.001 |
| eheals7~eheals8 | 0.47 | 0.035 | <0.001 |
|  |  |  |  |
| **Two-factor model** |  |  |  |
| F1 |  |  |  |
| eheals1 | 0.913 | 0.008 | <0.001 |
| eheals2 | 0.914 | 0.008 | <0.001 |
| eheals3 | 0.858 | 0.012 | <0.001 |
| eheals4 | 0.897 | 0.009 | <0.001 |
| eheals5 | 0.884 | 0.01 | <0.001 |
| F2 |  |  |  |
| eheals6 | 0.895 | 0.01 | <0.001 |
| eheals7 | 0.909 | 0.009 | <0.001 |
| eheals8 | 0.879 | 0.011 | <0.001 |
|  |  |  |  |
| **Revised two-factor model** |  |  |  |
| F1 |  |  |  |
| eheals1 | 0.916 | 0.008 | <0.001 |
| eheals2 | 0.918 | 0.008 | <0.001 |
| eheals3 | 0.828 | 0.014 | <0.001 |
| eheals4 | 0.873 | 0.011 | <0.001 |
| eheals5 | 0.894 | 0.01 | <0.001 |
| F2 |  |  |  |
| eheals6 | 0.916 | 0.01 | <0.001 |
| eheals7 | 0.594 | 0.043 | <0.001 |
| eheals8 | 0.84 | 0.015 | <0.001 |
| eheals3~eheals4 | 0.493 | 0.035 | <0.001 |
| eheals7~eheals8 | 0.331 | 0.044 | <0.001 |
