# Supplementary material for: Psychometric Validation and Cultural Adaptation of the Simplified Chinese eHealth Literacy Scale: Cross-Sectional Study
Source: J Med Internet Res. 2020 Dec 7;22(12):e18613. doi: 10.2196/18613 (PMC7752540; doi:10.2196/18613)
Supplement: Multimedia Appendix 3 [file jmir_v22i12e18613_app3.docx]

**Respondents’ mean SC-eHEALS score**

|  | SC-eHEALS score | p-value |
| --- | --- | --- |
| Sex |  |  |
| Male | 29.9 | 0.01 |
| Female | 28.5 |  |
| Age |  |  |
| ≤30 | 30.8 | 0.003 |
| 31-60 | 29.3 |  |
| ≥61 | 27.3 |  |
| Education |  |  |
| No/primary | 28.3 | 0.016 |
| Secondary/post-secondary | 28.5 |  |
| Tertiary or above | 30.3 |  |
| Family registry |  |  |
| Urban | 29.3 | 0.9 |
| Rural | 29.2 |  |
| Caregiver |  |  |
| No | 29.8 | 0.23 |
| Yes | 29.1 |  |
| Living status |  |  |
| Live alone | 29.3 | 0.1 |
| Live with family/others | 29.3 |  |
| Working status |  |  |
| Employed | 29.9 | 0.003 |
| Non-emloyed | 27.7 |  |
| Income level |  |  |
| ≤ 900 | 29.1 | 0.59 |
| 901 ~ 1800 | 27.9 |  |
| 1801 ~ 2700 | 29.1 |  |
| 2701 ~ 3800 | 29 |  |
| 3801 ~ 6400 | 29.9 |  |
| ≥ 6401 | 29.5 |  |
| BMI |  |  |
| BMI <23 | 29.4 | 0.5 |
| BMI ≥23 | 29.1 |  |
| Chronic condition |  |  |
| Yes | 29.9 | 0.01 |
| No | 28.5 |  |
| Self-reported health condition |  |  |
| Severe threat to life | 29.3 | 0.41 |
| Moderate threat to life | 28.5 |  |
| Mild threat to life | 28.9 |  |
| No threat to life | 29.8 |  |
