# Supplementary material for: Psychometric Validation and Cultural Adaptation of the Simplified Chinese eHealth Literacy Scale: Cross-Sectional Study
Source: J Med Internet Res. 2020 Dec 7;22(12):e18613. doi: 10.2196/18613 (PMC7752540; doi:10.2196/18613)
Supplement: Multimedia Appendix 4 [file jmir_v22i12e18613_app4.docx]

**Comparisons between IRT models**

|  | AIC | AICc | SABIC | HQ | BIC | logLik | X2 | df | p-value |
| --- | --- | --- | --- | --- | --- | --- | --- | --- | --- |
| RSM | 7851.606 | 7852.162 | 7865.743 | 7871.979 | 7903.837 | -3913.803 |  |  |  |
| PCM | 7848.38 | 7852.535 | 7887.256 | 7904.405 | 7992.017 | -3891.19 | 45.226 | 21 | 0.002 |
|  |  |  |  |  |  |  |  |  |  |
| PCM | 7848.38 | 7852.535 | 7887.256 | 7904.405 | 7992.017 | -3891.19 |  |  |  |
| GPCM | 7610.709 | 7616.863 | 7657.831 | 7678.618 | 7784.814 | -3765.354 | 251.671 | 7 | 0 |
|  |  |  |  |  |  |  |  |  |  |
| RSM | 7851.606 | 7852.162 | 7865.743 | 7871.979 | 7903.837 | -3913.803 |  |  |  |
| GPCM | 7610.709 | 7616.863 | 7657.831 | 7678.618 | 7784.814 | -3765.354 | 296.897 | 28 | 0 |
