# Supplementary figures and images for: Psychometric Validation and Cultural Adaptation of the Simplified Chinese eHealth Literacy Scale: Cross-Sectional Study
Source: J Med Internet Res. 2020 Dec 7;22(12):e18613. doi: 10.2196/18613 (PMC7752540; doi:10.2196/18613)

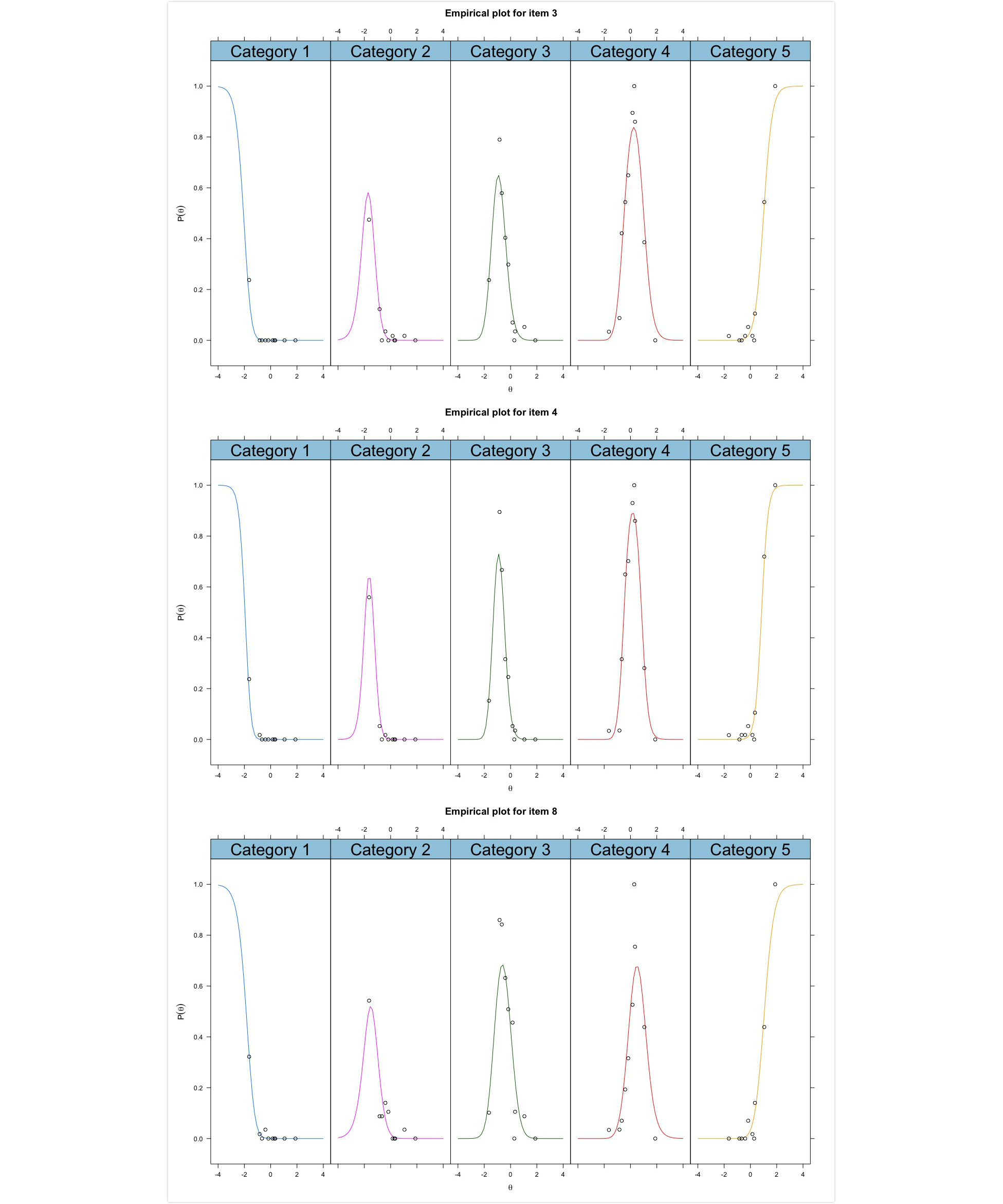

Supplement: Multimedia Appendix 5 [file jmir_v22i12e18613_app5.png]

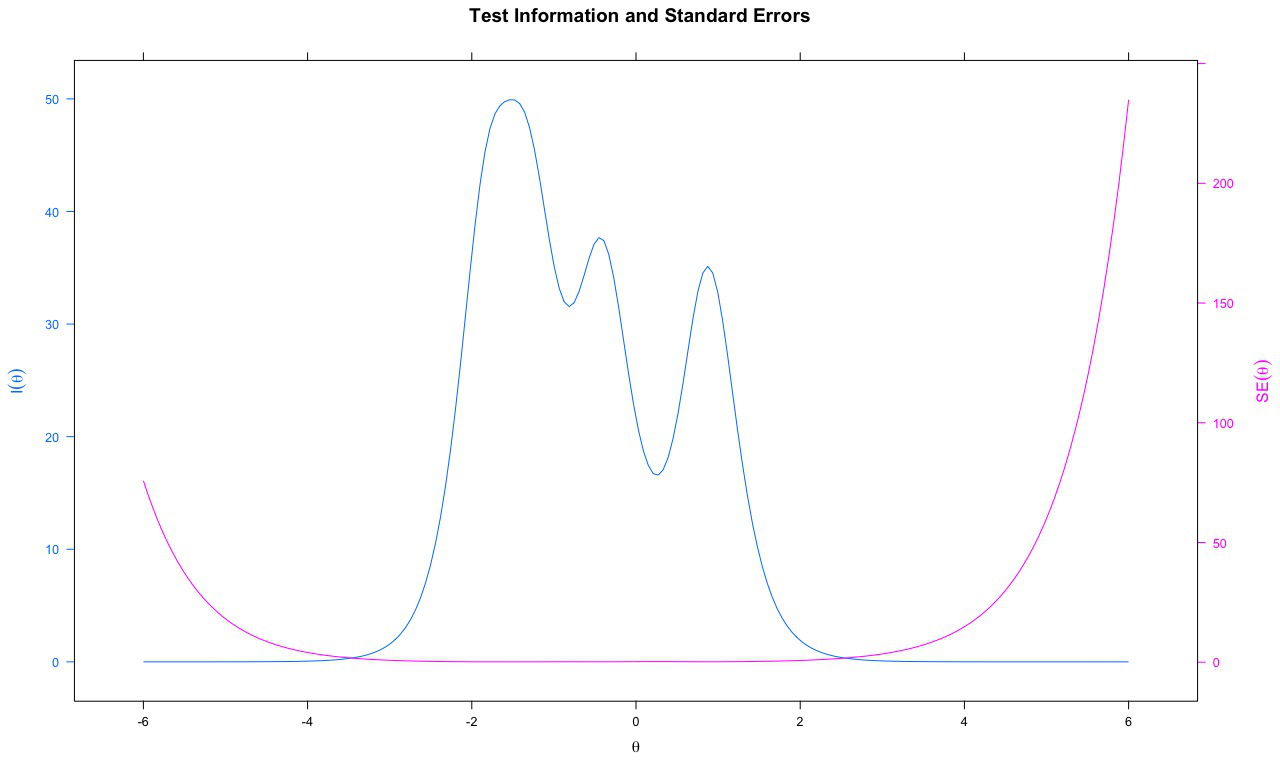

Supplement: Multimedia Appendix 6 [file jmir_v22i12e18613_app6.png]
