# Supplementary material for: Psychometric Validation and Cultural Adaptation of the Simplified Chinese eHealth Literacy Scale: Cross-Sectional Study
Source: J Med Internet Res. 2020 Dec 7;22(12):e18613. doi: 10.2196/18613 (PMC7752540; doi:10.2196/18613)
Supplement: Multimedia Appendix 7 [file jmir_v22i12e18613_app7.docx]

**Results of DIF analysis**

| item | chi12 | chi13 | chi23 | pseudo12.McFadden | pseudo13.McFadden | pseudo23.McFadden |
| --- | --- | --- | --- | --- | --- | --- |
| 1 | 0.614 | 0.8869 | 0.9184 | 0.0008 | 0.001 | 0.0001 |
| 2 | 0.5745 | 0.2229 | 0.1008 | 0.0009 | 0.0048 | 0.0039 |
| 3 | 0.7552 | 0.9662 | 0.9953 | 0.0005 | 0.0005 | 0 |
| 4 | 0.7755 | 0.4477 | 0.2024 | 0.0004 | 0.0031 | 0.0027 |
| 5 | 0.5717 | 0.8682 | 0.9318 | 0.0008 | 0.0009 | 0.0001 |
| 6 | 0.001 | 0.0055 | 0.6817 | 0.0117 | 0.0123 | 0.0006 |
| 7 | 0.7731 | 0.1272 | 0.0359 | 0.0003 | 0.0046 | 0.0043 |
| 8 | 0.1196 | 0.2134 | 0.4566 | 0.0028 | 0.0039 | 0.001 |

chi, Chi-squared test
