# Supplementary material for: Psychometric Validation and Cultural Adaptation of the Simplified Chinese eHealth Literacy Scale: Cross-Sectional Study
Source: J Med Internet Res. 2020 Dec 7;22(12):e18613. doi: 10.2196/18613 (PMC7752540; doi:10.2196/18613)
Supplement: Multimedia Appendix 8 [file jmir_v22i12e18613_app8.docx]

**The Chinese version of eHEALS**

我们想了解有关您使用互联网去获取健康资讯的经验。对于以下表述，请选择最符合您情况的选择。

|  |  | **非常**  **不同意** | **不同意** | **不确定** | **同意** | **非常**  **同意** |
| --- | --- | --- | --- | --- | --- | --- |
| 1 | 我知道什么样的健康资讯是可以从互联网上找到的 |  |  |  |  |  |
| 2 | 我知道网上可以从何处找到有用的健康资讯 |  |  |  |  |  |
| 3 | 我知道怎样在互联网上找到有用的资讯 |  |  |  |  |  |
| 4 | 我知道如何利用互联网去寻找我想知道的健康问题的答案 |  |  |  |  |  |
| 5 | 我知道如何利用我在互联网上找到的健康资讯去解决问题 |  |  |  |  |  |
| 6 | 我掌握必要的技能可以让我评估互联网上找到的资讯是否有用 |  |  |  |  |  |
| 7 | 我可以区分互联网上健康资讯质量的高低 |  |  |  |  |  |
| 8 | 我可以自信地使用互联网上找到的资讯去做出有关健康的决定 |  |  |  |  |  |
