# Supplementary material for: Psychometric Validation and Cultural Adaptation of the Simplified Chinese eHealth Literacy Scale: Cross-Sectional Study
Source: J Med Internet Res. 2020 Dec 7;22(12):e18613. doi: 10.2196/18613 (PMC7752540; doi:10.2196/18613)
Supplement: Multimedia Appendix 9 [file jmir_v22i12e18613_app9.docx]

**Comparisons between this study and previous validation studies**

|  | **Chang & Schulz** | **Ma & Wu** | **Zibrik et al.** | **Guo et al.** | **This study** |
| --- | --- | --- | --- | --- | --- |
| **Publication language** | English | English | English | Chinese | English |
| **Sample characteristics** | | | | | |
| **Respondents** | Patients with chronic condition | General public | Immigrant Chinese and Punjabi senior populations | High school student | Inpatient patient |
| **Setting** | One hospital | One rural county | Canada, British Columbia | Three high schools from three cities | Five hospitals from five cities |
| **Sample source** | Developed area | Underdeveloped area | Not in China | Developed area | Both developed and underdeveloped area |
| **Data collection time** | 2015-2016 | 2017 | 2013 and 2014 | 2012 | 2019-2020 |
| **Format** | Face-to-face | Face-to-face | Face-to-face | Face-to-face | Face-to-face |
| **Sample size** | 352 | 543 | 338 | 110 | 574 |
| **Psychometric analysis** | | | | | |
| **Reliability** | •   Cronbach’s alpha  •   Split-half | •   Cronbach’s alpha | No | •   Cronbach’s alpha | •   Cronbach’s alpha  •   McDonald’ omega  •   Split-half |
| **Item statistics** | Yes | Yes | Yes | Yes | Yes |
| **Convergent validity** | Yes | No | No | No | Yes |
| **Construct validity** | •   Confirmatory factor analysis | •   Principal components analysis  •   Confirmatory factor analysis | No | •   Exploratory factor analysis | •   Confirmatory factor analysis  •   Exploratory factor analysis |
| **Known-group validity** | No | No | No | No | Yes |
| **Criterion validity** | No | Yes | No | No | No |
| **IRT analysis** | No | •   Graded response model | No | No | •   Partial credit model  •   General particle credit model  •   Rating scale model |
| **DIF** | No | No | No | No | Yes |
